# Hippocampal connectivity with sensorimotor cortex during volitional finger movements: Laterality and relationship to motor learning

**DOI:** 10.1371/journal.pone.0222064

**Published:** 2019-09-19

**Authors:** Douglas D. Burman

**Affiliations:** Department of Radiology, NorthShore University HealthSystem, Evanston, Illinois, United States of America; Texas Tech University, UNITED STATES

## Abstract

Hippocampal interactions with the motor system are often assumed to reflect the role of memory in motor learning. Here, we examine hippocampal connectivity with sensorimotor cortex during two tasks requiring paced movements, one with a mnemonic component (sequence learning) and one without (repetitive tapping). Functional magnetic resonance imaging activity was recorded from thirteen right-handed subjects; connectivity was identified from sensorimotor cortex correlations with psychophysiological interactions in hippocampal activity between motor and passive visual tasks. Finger movements in both motor tasks anticipated the timing of the metronome, reflecting cognitive control, yet evidence of motor learning was limited to the sequence learning task; nonetheless, hippocampal connectivity was observed during both tasks. Connectivity from corresponding regions in the left and right hippocampus overlapped extensively, with improved sensitivity resulting from their conjunctive (global) analysis. Positive and negative connectivity were both evident, with positive connectivity in sensorimotor cortex ipsilateral to the moving hand during unilateral movements, whereas negative connectivity was prominent in whichever hemisphere was most active during movements. Results implicate the hippocampus in volitional finger movements even in the absence of motor learning or recall.

## Introduction

Does the hippocampus play a role in executing volitional finger movements? There are several reasons to suspect it might. Although often assumed to reflect its known role in memory function [1–4], the hippocampus shows motor activity [5], especially during motor sequence learning [6–12]. Furthermore, the hippocampus has been implicated in the generation of theta waves [13, 14], which enhances motor performance [15–17]. Finally, low-threshold electrical stimulation of the hippocampus induces seizures [18], suggesting an intimate interaction with the motor system. These considerations suggest the hippocampus is intimately involved in movements, particularly volitional movements.

Paced movements require volitional movements, and the primary motor cortex is necessary for volitional movements of individual fingers [19, 20]. Short fiber tracts connect postcentral with precentral regions [21], providing sensory feedback required for accurate performance. The psychophysiological interaction (PPI) technique for studying connectivity allows us to study task-specific hippocampal influences on sensorimotor cortex (SMC) during these movements. In this technique, the hemodynamic signal is deconvolved to the underlying neural activity [22], then *interaction effects* between motor- and non-motor activity in the seed region (hippocampus) demonstrate differential effects on the magnitude or sign of response in the target region. This technique demonstrates the directional influence of connectivity on a moment-to-moment basis [23], important since the temporal pattern of hippocampal activity carries information [24–26].

This study explores the laterality and memory requirements of hippocampal-SMC connectivity during paced movements. Anticipatory motor responses confirmed a cognitive role during both sequence learning and paced, repetitive tapping. Hippocampal sources of connectivity were bilateral, whereas the laterality of SMC connectivity depended both on the task and seed; nonetheless, connectivity with the SMC hand representation increased during both tasks, suggesting connectivity was not limited to memory functions.

## Materials and methods

### Subjects

Thirteen right-handed adults from the Chicago metropolitan area participated in the study (ages 249, mean = 42.3, five females). The nature of experimental procedures were explained to each subject before obtaining written consent; consent procedures complied with the Code of Ethics set forth in the Declaration of Helsinki, and were approved by the Institutional Review Board Board at the NorthShore University HealthSystem / Research Institute. Consented subjects had no history of the following: a previous concussion, psychiatric illness, learning disability, attention deficit disorder, abnormal physical or cognitive development that would affect educational performance, central neurological disease (or structural pathology), or neurosurgical intervention.

### Experimental task

Each subject performed a visual/motor task with a total duration of 6m 4s, consisting of 6 cycles of a specified sequence of visual and motor conditions. Each cycle included a block of sequential tapping and a block of repetitive tapping, separated by a passive visual condition. The task ended with 10s of passive fixation on a central cross.

**Fig 1:**
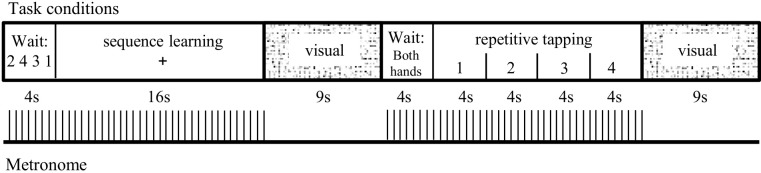
One cycle of the visual / motor task. Including their instruction screens, two motor conditions alternated with a visual condition; this sequence repeated for a total of six cycles. Motor conditions were accompanied by a metronome ticking at two beats per sec for pacing finger movements.

The timeline for a single cycle is diagrammed in Fig 1. Each cycle began with a 4s instruction screen, when a metronome ticked through sound-attenuated headphones [MR Confon Mk II] at 2 beats/sec as a 4-digit sequence appeared onscreen to specify the correct order of button presses. Once the digits were replaced with a cross, the subject pressed the remembered sequence of buttons synchronously with the metronome, repeating the 4-button sequence throughout the 16s block (8 repetitions). A visual block followed; a circular checkerboard pattern flickered at 4Hz for 9s, while subjects fixated the center of the pattern and refrained from moving.

After the visual block, another 4s instruction screen instructed the subject to tap the same finger with both hands on cue. Once the instruction screen was replaced with the number '1,' the subject tapped the index finger from both hands in synchrony with the metronome; every 4s, the onscreen number increased by one, and the subject changed finger. Each finger tapped for 8 repetitions during the 16s repetitive tapping block; the cycle ended with another visual block.

With this design, each motor condition (with its instruction screen) was preceded and followed by a period of rest. Button presses during sequence learning were performed with the dominant (right) hand, with a new sequence specified at the beginning of the first 4 cycles; the first 2 sequences were repeated at the end to test for motor recall. Button presses were recorded from the right hand during both sequence learning and repetitive tapping.

### Behavioral analysis and terminology

Four buttons, arranged horizontally on the response pad, were each pressed by a different finger, labelled sequentially as 1 (index finger) through 4 (little finger). Occasionally, button contact was not firm enough to be recorded, resulting in missing data; however, all recorded button presses were consistent with 100% accurate performance for both tasks.

In the sequence learning block, a subject repeatedly tapped a 4-button sequence in synchrony with the metronome, beating at 500ms intervals. With four finger movements during each sequence, several behavioral measures were possible. In this study, three behavioral measures were calculated for each repetition: the mean stimulus-response asynchrony (the time interval between the metronome beat and the button press), the mean intertap interval, and the mean precision of intertap intervals (i.e., the absolute difference between 500ms and the intertap interval). Each mean value represented 3 or 4 measurements from a single sequence, so the standard deviation was calculated as a measure of the individual subject's response variability.

The intertap interval can be used as an example of our general method for visualizing and analyzing behavioral data. The mean intertap interval across all subjects was plotted (i.e., the group mean of the individuals' mean intertap intervals), and a paired t-test evaluated whether the intertap intervals during the final two rehearsals significantly differed from the initial two rehearsals of the sequence. This test used *between-subject* variability to evaluate group changes in the mean intertap interval; if each subject initially had a mean intertap interval of 500ms, a consistent drop of 10ms in this mean group value would be significant, even if each subject had a standard deviation of 100ms.

The mean standard deviation across all subjects was also plotted (i.e., the group mean of the individuals' standard deviations). A paired t-test evaluated whether the variability in intertap intervals during the final two rehearsals differed from the initial two rehearsals. This test evaluated changes in *within-subject* variability; if each subject began with a standard deviation of 100 ms, consistent changes to 80 ms would be significant, even if the mean intertap interval for the group remained the same at 500 ms.

Stimulus-response asynchrony differentiated between reflexive movements (consistent short-latency responses following stimulus presentation) vs. intentional movements under cognitive control (anticipatory responses that precede the metronome sounds). Changes in intertap intervals and their precision identified learning effects resulting from rehearsal.

To compare behavioral performance between motor tasks, the same behavioral measures and statistical tests were additionally applied to 4-tap groupings during the repetitive tapping task. A paired t-test identified task differences in performance, both for the initial and final pair of rehearsals.

### MRI data acquisition

Images were acquired using a 12-channel head coil in a 3 Tesla Siemens scanner (Verio). Visual stimuli projected onto a screen (Avotec Silent Vision) were viewed via a mirror attached to the head coil, and behavioral responses were recorded by Eprime [Psychology Software Tools, Inc.] from an optical response box (Current Designs, Philadelphia, PA). Blood-oxygen level dependent (BOLD) functional images were acquired using the echo planar imaging (EPI) method, using the following parameters: time of echo (TE) = 25 ms, flip angle = 90°, matrix size = 64 x 64, field of view = 22x22 cm, slice thickness = 3 mm, number of slices = 32; time of repetition (TR) = 2000 ms; and the number of repetitions = 182. A structural T1 weighted 3D image (TR = 1600 ms, TE = 3.46 ms, flip angle = 9°, matrix size = 256 x 256, field of view = 22x22 cm, slice thickness = 1 mm, number of slices = 144) was acquired in the same orientation as the functional images.

### fMRI data processing

Data was analyzed using SPM8 software (http://www.fil.ion.ucl.ac.uk/spm). Images were spatially aligned to the first volume to correct for small movements. The last cycle of the visuomotor task was removed from one subject’s fMRI data, as repeated head movements approached unacceptable limits; after its removal, no run exceeded 3mm displacement along the x, y or z dimension, with maximal RMS movement for any subject limited to 1.5mm (mean = 0.850 + 0.291mm). Sinc interpolation minimized timing-errors between slices; functional images were coregistered to the anatomical image, normalized to the standard T1 Montreal Neurological Institute (MNI) template, and resliced as 4mm isometric voxels.

Processing parameters suitable for identifying movement-related activation in sensorimotor cortex differed from those optimized to examine hippocampal connectivity, which will be described in a later section. To identify task-related activation, data were smoothed with a 10mm isotropic Gaussian kernel and filtered with a high pass cutoff frequency of 128s. Conditions of interest were specified for sequence learning, visual, and each of the four fingers moving during the repetitive tapping block; the latter four conditions were combined to identify brain activity across the entire repetitive tapping block. Blocks were modeled for analysis using a canonical hemodynamic response function (HRF).

In this analysis, global normalization scaled the mean intensity of each brain volume to a common value to correct for whole brain differences over time. A parameter estimate of the BOLD response to each condition was generated; motor activation was identified by contrasting mean BOLD responses to motor vs. visual conditions. In a second-level analysis, BOLD contrasts from individual subjects during the motor memory and repetitive tapping conditions were entered into a two-cell ANOVA, and group analysis identified activation from individual tasks (sequence learning or repetitive tapping), task differences (sequence learning vs. repetitive tapping), and global conjunction (common activation during sequence learning and repetitive tapping, after accounting for task differences in variability). Analyses used an intensity threshold of p<0.05 with a family-wise error (FWE) correction for multiple comparisons, applied to a sensorimotor region of interest (ROI). This ROI was derived from the WFUPickatlas toolbox, and was specified as the overlap between TD and aal atlas labels for post- plus precentral gyrus.

### Laterality analysis

Laterality was assessed using the following formula:

$$\text{Laterality}\;\text{Index}\;\left(\text{LI}\right)=\frac{\left(\text{voxels}\;\text{on}\;\text{the}\;\text{right}-\text{voxels}\;\text{on}\;\text{the}\;\text{left}\right)}{\left(\text{voxels}\;\text{on}\;\text{the}\;\text{right}+\text{voxels}\;\text{on}\;\text{the}\;\text{left}\right)}$$

This provided a continuous variable, ranging from LI = -1 (activation or connectivity limited to the left hemisphere) to LI = +1 (activation or connectivity limited to the right hemisphere).

Laterality was used to assess hemispheric differences in activation for a unilateral condition (sequence learning), hemispheric differences in activation for a bilateral condition (repetitive tapping), and hemispheric differences in sensorimotor connectivity during both conditions. These comparisons served to differentiate between SMC laterality differences attributable to unimanual vs. bimanual movements.

### Psychophysiological interactions (PPI)

#### Preprocessing

Processing parameters optimized for hippocampal activity differ from those that identified task-related activation in sensorimotor cortex. To study hippocampal-SMC connectivity, this study applied a smaller smoothing kernel (6mm instead of 10mm), did not apply global scaling for intensity, and used a single condition of interest for movements during the repetitive tapping task. Preprocessing did not otherwise differ from the methodology described above.

Connectivity analysis was carried out using psychophysiological interactions [22], modified to account for individual variability in connectivity [27]. A total of 156 voxels were identified from the left (78) and right hippocampus (78) of the normalized brain, as delimited by the aal atlas in the WFU PickAtlas toolbox (http://fmri.wfubmc.edu/software/PickAtlas). Coordinates for these voxels are specified in S1 and S2 Tables. Labelling of individual voxels began at the posterior/superior/medial edge of the hippocampus in each hemisphere (L or R), moving systematically laterally (1 to 5), inferior (11 to 31), and anterior (111 to 911).

A contrast was selected and specified to create eigenvariates for all conditions in the statistical model at each hippocampal voxel (assured by selecting a p-value threshold of 1). An interaction term specified a greater effect of motor activity in the seed than during the visual condition; after adjustments for regional differences in timing and baseline activity, a regression analysis showed the magnitude of the BOLD signal that correlated with this interaction term elsewhere in the brain. In this approach, connectivity with SMC was quantified at each voxel, with the net effect of a hippocampal region on SMC identified from the mean of connectivity values generated across voxels.

#### Seed selection

Appropriate selection of seed regions for connectivity analysis is critical [28], but because the temporal pattern of hippocampal activity affects information processing elsewhere [29–33], identifying areas of increased hippocampal activity through activation analysis may be inadequate. We therefore adopted two approaches. The first approach was structural. The hippocampus was divided into a 3x3 matrix, with net connectivity evaluated for each region. The threshold for significance was p<0.05 after a family-wise error (FWE) correction for multiple voxel comparisons within the hand representation, plus an additional correction for multiple comparisons of 9 structural seeds. Structural seeds were labelled by their position within the matrix (A to C from anterior to posterior, 1 to 3 from medial to lateral) and the sign of connectivity (positive or negative). The location of these seeds (and their component voxels) can be seen in S1 Fig.

The second approach was functional. A functional seed was identified from the left and right hippocampus for both sequence learning (“memseed”) and repetitive tapping (“tapseed”). Selection of functional seeds was restricted to voxels within (or adjacent to) a structural seed showing significant connectivity; the single voxel was selected that showed maximal connectivity within the SMC mask. (The location of functional seeds can be seen in S2 Fig.) The SMC mask did not limit findings to the hand region; in fact, the maximum for both positive and negative connectivity was most often outside the hand representation, varying between pre- and postcentral gyrus and even between hemispheres (S3 Table). By reflecting individual variability in functional localization, functional seeds provide a better estimate of the magnitude and extent of hippocampal influence on SMC activity.

#### Group analysis

Beta estimates of connectivity from each subject's left and right hippocampus were entered into a two-cell ANOVA, using the hand representation as the ROI and an intensity threshold of p<0.05 with FWE correction. Using the processing parameters optimized for connectivity analysis, the ROI consisted of the bilateral SMC region activated during finger movements in either task (sequence learning or repetitive tapping). This ROI reflected our primary hypothesis: hippocampal connectivity with the finger region of SMC appeared preferentially during volitional finger movements. SMC connectivity was mapped from each hippocampus; in addition, global analysis of connectivity from both hippocampi identified joint effects.

### Testing for cortical selectivity

The hypergeometric distribution [34] tested the cortical selectivity of connectivity by calculating the probability that the observed number of connectivity voxels reaching statistical significance overlapped the hand representation in the left or right SMC by chance. Cortical selectivity was demonstrated by a probability less than five percent (p<0.005 before correcting for multiple comparisons).

This approach assumes the connectivity threshold could be exceeded anywhere within SMC, i.e., the interaction term between motor and visual conditions in the hippocampus does not merely reproduce the activation analysis used to identify the SMC hand representation. In fact, these methods differ markedly. Activation analysis identified where the mean SMC activity during motor blocks exceeded the mean activity during visual blocks; by contrast, the interaction term used in PPI analysis reflected the temporal pattern of hippocampal activity throughout both blocks.

### Independence of activation and connectivity

**Fig 2:**
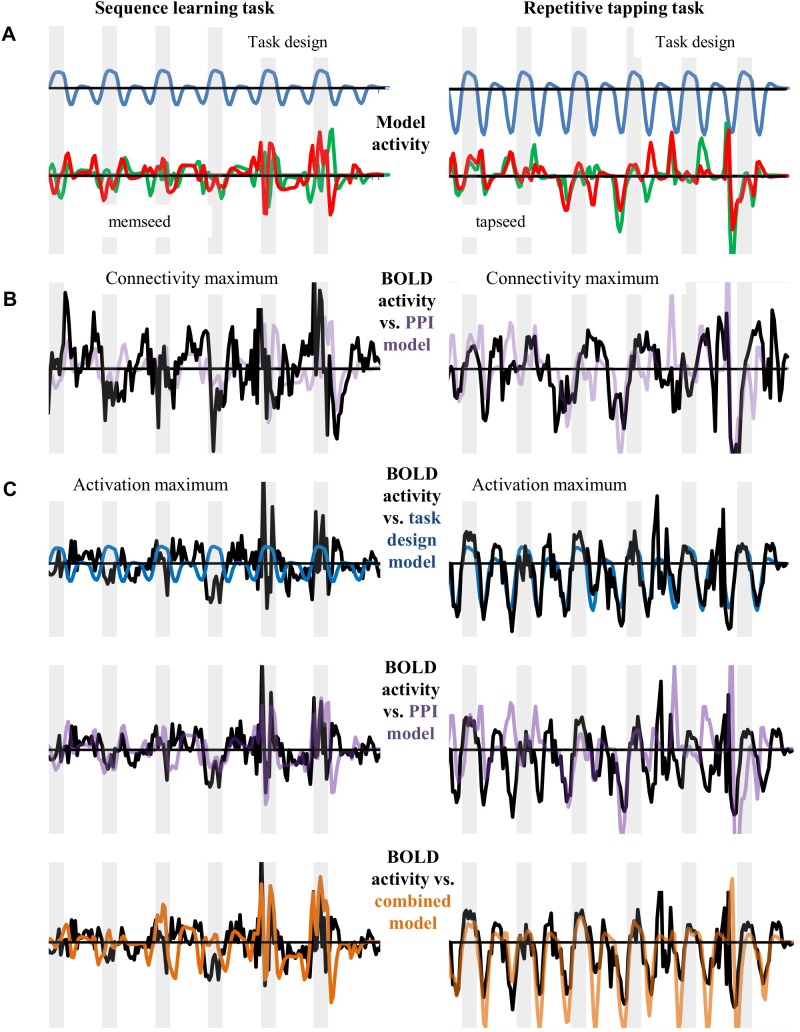
Independence of activation and connectivity analysis. (A) Predicted patterns of BOLD activation (top, blue), and connectivity from the left and right hippocampal seeds (bottom, green and red, respectively). (B) BOLD activity observed at the SMC connectivity maximum (black) compared with activity predicted from the sum of the activity from the left and right hippocampal seeds (purple). (C) BOLD activity observed at the SMC activation maximum (black) compared to the predicted pattern for activation (top, blue), the sum of activity from left and right hippocampal seeds (middle, purple), and the sum of activation plus hippocampal activity (bottom, orange).

Fig 2 shows differences between activation and PPI connectivity in one subject, illustrating their independence. During activation analysis, a motor condition (sequence learning or repetitive tapping) was contrasted with the passive visual condition; based upon the block design, the predicted BOLD response is the same across all 6 task cycles (Fig 2A, blue waveform at top). For each task, the BOLD response in SMC predicted from the task design was elevated above baseline during the motor block (shaded gray), and depressed below baseline during the visual condition. The BOLD response in SMC predicted by PPI connectivity analysis, on the other hand, was based on hippocampal seed activity, after accounting for regional differences in timing and amplitude of neural activity. The green and red traces at the bottom of Fig 2A show this subject’s predicted BOLD response based on connectivity, derived from activity in the left and right hippocampal seeds, respectively. Note that the predicted pattern of activity for connectivity changes across cycles, and that predicted increases in the BOLD response above baseline often do not coincide with those predicted for activation from the task design. Indeed, SMC BOLD responses predicted from activity in the left and right hippocampal seeds show greater similarity to each other than to the response predicted for activation from the task design.

The BOLD signal observed at the SMC *connectivity maximum* (Fig 2B, black line) approximated the sum of BOLD signals predicted from combined activity in the left and right seeds (purple line). In the sequence learning task (left), the peaks and valleys at this right SMC voxel generally did not coincide with those predicted from activation analysis; although connectivity was significant at this voxel, activation was not. In the repetitive tapping task (right), the BOLD signal at the connectivity maximum again approximated the sum of BOLD signals predicted from activity in the left and right seeds; due to sufficient overlap with the pattern predicted from the task design, there was also significant activation.

For both tasks, the BOLD signal at the *activation maximum* approximated the response predicted from the task design (Fig 2C, top, blue line), but also the response predicted from connectivity analysis (Fig 2C, middle, purple line). However, the BOLD signal most closely mirrored the response predicted from the sum of activation and connectivity analyses (Fig 2C, bottom, orange line).

Thus, the pattern of BOLD activity in a subject’s SMC could reflect the pattern of seed activity predicted from connectivity analysis, activation analysis, or both.

## Results

### Behavioral performance during motor tasks

**Fig 3:**
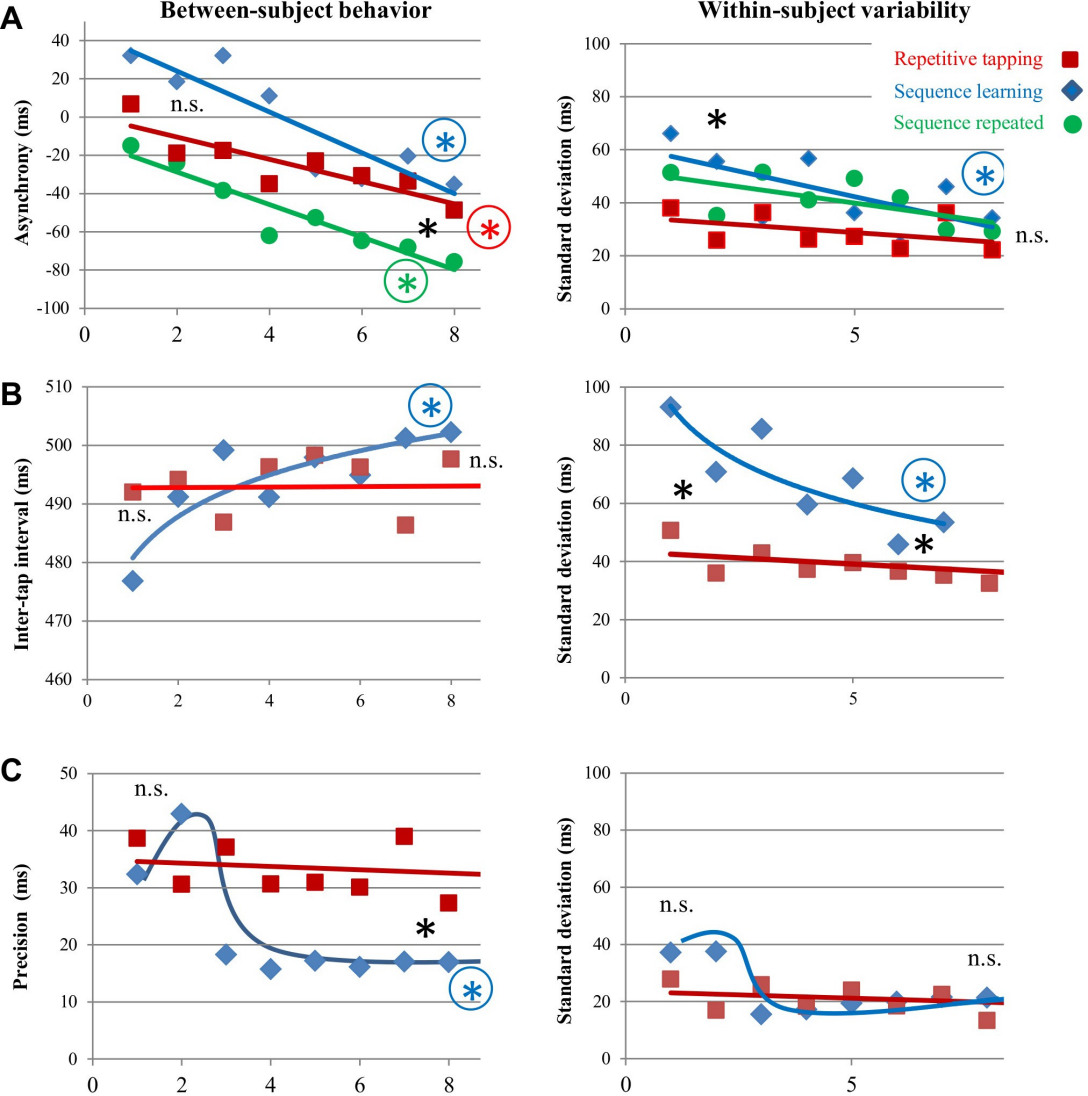
Rehearsal effects on motor performance during sequence learning and repetitive tapping. (A) Changes in stimulus-response asynchrony across rehearsals. Negative values for asynchrony represent anticipatory responses, which developed across rehearsals for all motor conditions; variability represents standard deviations for 4-note groupings among individuals. Colored encircled asterisks indicate a significant difference from the first to last pair of repetitions during a task; a plain black asterisk at the beginning or end of rehearsals indicates a significant difference between task conditions (p<0.05). (B) A change in intertap intervals was observed across rehearsals only in the sequence learning task, accompanied by a decrease in variability. (C) A change in precision was observed across rehearsals only in the sequence learning task.

Stimulus-response asynchrony, intertap intervals, and precision of intervals were characterized for both sequence learning and repetitive tapping tasks (Fig 3, see also Tables 1 and 2). The left column in Fig 3 shows group changes in performance across rehearsals, calculated from mean values for all subjects; the right column shows the mean standard deviation among individuals.

**Table 1:** Group rehearsal effects during sequence learning and repetitive tapping.

Group rehearsal effects (ms)								
Performance measure	Sequence learning			Repetitive tapping			Task differences (p-value)	
	Start	End	p-value	Start	End	p-value	Start	End
Asynchrony: first sequence	32.0±24.5	-35.2±18.8	.043*	6.7 ± 21.5	-48.8±18.3	.038*	.132	.739
repeat sequence	-14.9±29.2	-75.7+19.4	< .001*	-20.0±24.2	-59.2±20.7	.036*	.886	.041*
Intertap intervals	476.8±8.9	502.2±5.6	.048*	492.0±9.4	497.6±4.4	.925	.222	.083
Precision	32.4±6.8	21.3±3.6	.009*	38.7±8.0	27.3±5.4	.617	.730	.001*

Group effects reflect mean performance in timing across subjects. Behavioral measures were compared statistically during the first and last pair of rehearsals; start and end values represent the mean ± standard error across all subjects from first and eighth repetition of 4-note sequences.

* p<0.05 in a paired t-test comparing the first to last pair of trials.

**Table 2:** Individual variability during sequence learning and repetitive tapping.

Individual variability (SD on tapping groups of 4)								
Performance measure	Sequence learning			Repetitive tapping			Task differences (p-value)	
	Start	End	p-value	Start	End	p-value	Start	End
Asynchrony: first sequence	66.0 ± 14.2	34.3 ± 5.7	.016*	37.8±11.9	23.2±4.2	.152	.043*	.076
repeat sequence	51.4 ± 14.2	29.2 ± 5.7	.087	34.2±12.0	23.1±6.0	.093	.237	.583
Intertap intervals	93.1 ± 18.8	65.2 ± 16.2	.031*	36.0±8.3	37.3±9.9	.113	.027*	.006*
Precision	37.1 ± 6.0	21.3 ± 6.1	.080	25.2±8.0	20.2±6.4	.902	.236	.208

Individual effects reflect within-subject variability, as measured by the standard deviation from 4-tap groupings. Behavioral measures were compared statistically during the first and last pair of rehearsals.

* p<0.05 in a paired t-test comparing the first to last pair of trials.

Fig 3A shows the effects of rehearsal on stimulus asynchrony (i.e., the time interval between the metronome and button presses). The desired pacing of finger tapping was set by a metronome during the instruction period. Finger movements during the movement task began synchronous or slightly after the metronome (left), but significantly anticipated the metronome with rehearsal (negative values). Repetitive tapping (red) and sequence learning (blue) generated similar anticipatory responses after 8 rehearsals, but an additional 8 rehearsals on a learned sequence generated greater asynchrony (green). Individual *variability* in asynchrony was initially greater during sequence learning, but differences were eliminated over 8 rehearsals as performance of a learned sequence became easier (right).

Learning effects were identified from rehearsal-related changes in intertap intervals and their precision (Fig 3B and 3C). Learning effects were observed during sequence learning but not the repetitive tapping task; the intertap interval during sequence learning significantly improved towards the paced interval of 500 ms, while its variability dropped. The precision of intertap intervals also improved during sequence learning (i.e., how much intertap intervals within a 4-key sequence differed from 500 ms), dropping below that observed during repetitive tapping.

### SMC activation during motor tasks

**Fig 4:**
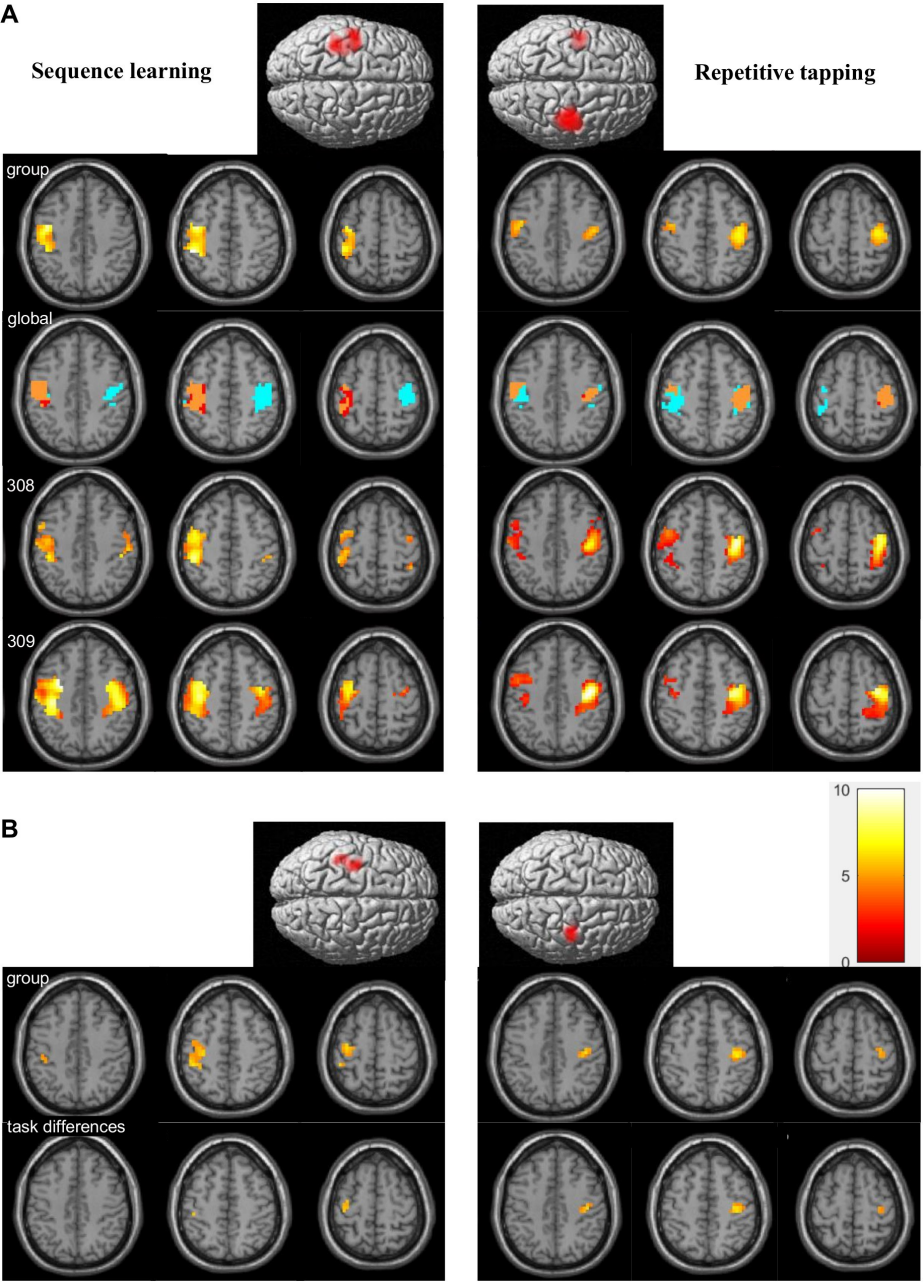
Sensorimotor activation during performance of motor tasks. (A) Group analysis (top) showed unilateral activation in left sensorimotor cortex during performance of the unimanual sequence learning task, and bilateral activation during the bimanual repetitive tapping task. Global analysis for the group expanded the area of demonstrable activation; in this analysis, subthreshold activation revealed through global analysis is shown in cyan, with overlap between global and individual task activation shown in orange. Individual analysis (subjects 308–309) verified bilateral activation for both tasks. (B) Task activation with a smaller (6mm) smoothing kernel used for connectivity analysis. The area of activation was reduced in both tasks, with the area of detectable activation during repetitive tapping limited to the right SMC. Direct task comparisons revealed greater left SMC activation during sequence learning and greater right SMC activation during repetitive tapping. Threshold for both individual and group activation was p = 0.05 with FWE correction for multiple comparisons. Results in this and subsequent figures are displayed using the neurological convention (left display = left side of brain).

SMC was activated during both sequence learning and repetitive tapping (Fig 4A and Table 3). Reflecting unimanual performance by the right hand, SMC activation during sequence learning was limited to the left hemisphere during group analysis (LI = -0.98), but not during individual analysis (mean LI = -0.47±0.33). By contrast, group activation during repetitive tapping was right-dominant (LI = 0.62), whereas activation during individual activation showed equal dominance (mean LI = 0.08±0.41), reflecting bimanual performance. Thus, group analysis of individual tasks poorly reflected the extent of motor activity present among individuals.

**Table 3:** Location of group sensorimotor cortical activation.

Task	Hemisphere	Cluster size	Z-score (peak)	Peak Coordinates
*Smoothing kernel = 10mm*				
Sequence learning	left	227	6.08 5.69 5.67	(-42,-36,54) (-42,-4,46) (-38,-8,54)
Repetitive tapping	right	157	5.04 5.01	(42,-20,54) (46,-16,50)
	left	71	4.69 4.27	(-42,-4,46) (-50,0,38)
Combined (global analysis)	left	165	5.82 5.65 5.07	(-46,-36,54) (-42,-4,46) (-50,0,38)
	right	168	5.16 5.05 5.00	(46,-32,50) (46,-16,50) (42,-24,54)
*Smoothing kernel = 6mm*				
Sequence learning	left	87	5.15	(-42,36,58) (-34,-16,58) (-30,-24,54)
Repetitive tapping	right	54	4.90	(34,-20,58) (42,-20,50)
Combined (global analysis)	right	52	4.80	(42,-20,50) (34,-20,58)
Task comparison				
sequence > repetitive	left	13	4.07	(-42,-24,58) (-34,12,62)
repetitive > sequence	right	40	4.78	(34,-20,58) (42,-20,50)

Activation clusters within the sensorimotor mask, surviving an intensity threshold of p = 0.05 with a family-wise error correction and extent threshold of 20. Peak locations are specified in MNI coordinates.

A more sensitive method for identifying the extent of motor activity during group analysis involved global (conjunctive) analysis of both motor conditions (Fig 4B). By revealing activation that was otherwise subthreshold, global analysis extended the area of group activation (cyan, see especially the right SMC for sequence learning). Global analysis of group activation better reflected the full extent of activation observed during individual analyses. Global conjunctive analysis without a mask demonstrated additional activation in the supplementary motor cortex and cerebellum, plus auditory activation from the metronome in the superior temporal gyrus (S4 Table).

Although not optimal for detecting the full extent of activation, a smoothing kernel of 6mm was applied to examine SMC connectivity from hippocampal seed activity. SMC activation was more localized under these conditions; for example, activation during repetitive tapping was restricted to right SMC, despite bimanual movements. Task comparisons showed each hemisphere was preferentially activated by a different task. SMC activation in both hemispheres (left SMC from sequence learning, right SMC from repetitive tapping) provided the ROI used for connectivity analysis.

### Hippocampal sensorimotor connectivity

**Fig 5:**
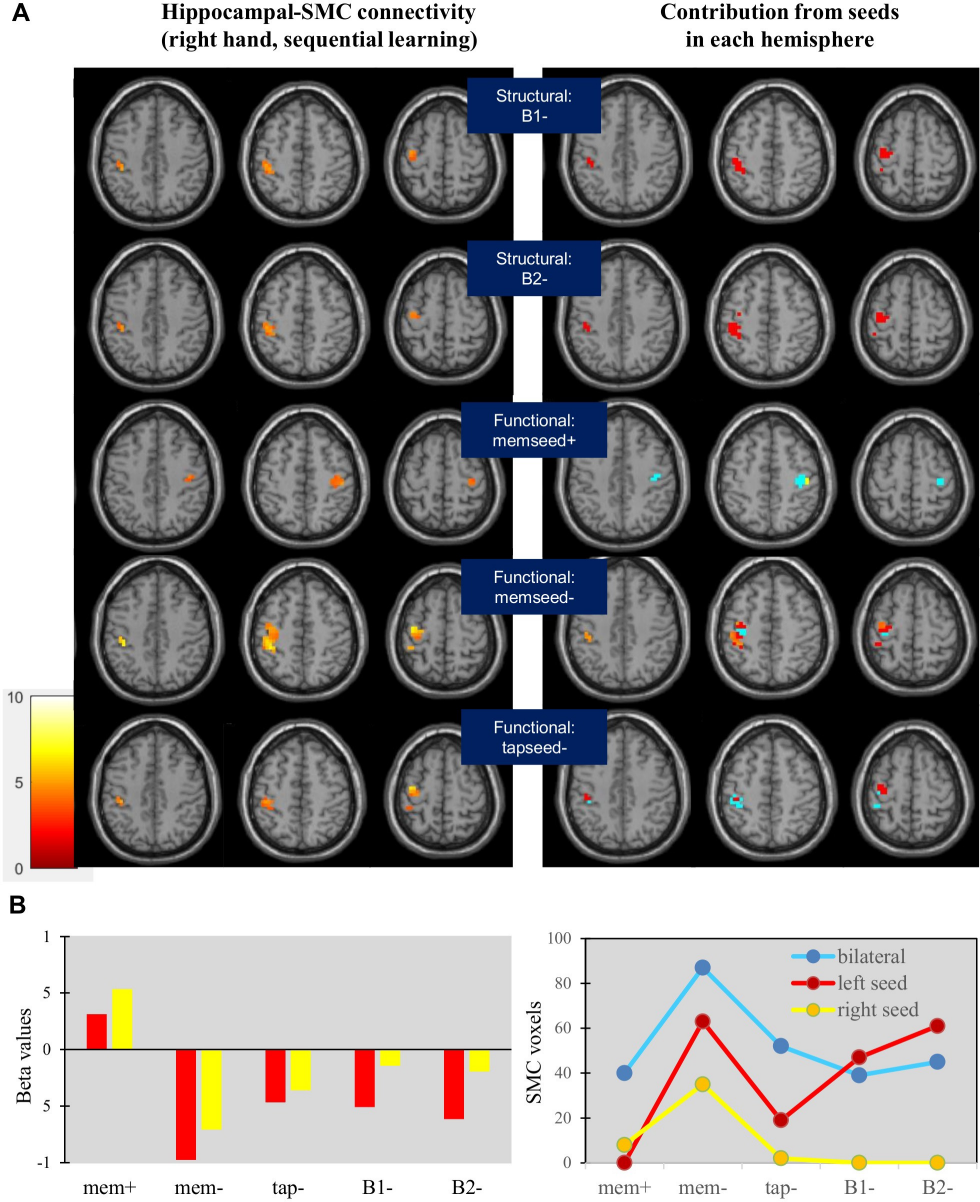
Sensorimotor connectivity of structural and functional seeds during sequential learning. (A) Negative connectivity with the left SMC was generated from three structural seeds (A2, B1, and B2) and a functional seed selected from each motor task; positive connectivity with the right SMC was generated from an anterior functional seed (left column, memseed+). Connectivity from the left hippocampus was more prevalent than from the right (right column). (B) Beta values for connectivity (left) and the total number of SMC voxels with significant connectivity generated from seeds in each hemisphere (right) indicate a role for both the left and right hippocampus. Connectivity from global analysis of both could be greater than connectivity from the left or right hippocampus by itself.

Hippocampal connectivity was evaluated within the SMC hand representation. In the sequence learning task, three structural seeds (A2, B1, and B2) generated negative connectivity with the left SMC (Fig 5A and Table 4). The A2 seed also generated a small area of positive connectivity with the right SMC; identifying positive and negative functional seeds within this hippocampal region revealed that different hippocampal voxels generated connectivity with the left or right SMC, but not both. The left hippocampus often played a greater role, but analysis of beta estimates and global analysis showed that the left and right hippocampus both contributed to SMC connectivity (Fig 5B).

**Table 4:** Connectivity clusters within sensorimotor cortex during group analysis.

Task	Seed	Hemisphere (SMC)	Region	Cluster size	Z-score (peak)	Peak Coordinates
Sequence learning	Structural: B1-	left	postcentral	29	4.18	(-34,-36,50) (-42,-28,54)
		left	precentral	10	4.64	(-34,-12,66)
	Structural: B2-	left	postcentral	32	4.09	(-42,-28,54)
		left	precentral	19	3.86	(-42,-28,58)
	Functional: memseed+	right	precentral postcentral	40	4.03	(46,-24,54) (42,-12,50) (30,-20,58)
	Functional: memseed-	left	precentral postcentral	87	5.01	(-34,-12,66) (-38,-36,58) (-38,-32,46)
	Functional: tapseed-	left	postcentral	33	4.21	(-38,-28,46) (-42,-28,54) (-38,-12,62)
		left	precentral	19	4.76	(-38,-12,62)
Repetitive tapping	Structural: A2-	right	precentral	44	4.28	(38,-24,46) (38,-28,58) (34,-16,54)
	Structural: A3-	right	precentral	42	4.13	(42,-24,46) (42,-24,58) (38,-16,58)
	Structural: B3-	right	precentral	22	3.91	(38,-24,46) (38,-28,58) (34,-16,54)
	Functional: memseed-	right	precentral	52	4.50	(42,-24,46) (34,-24,62)
	Functional: tapseed-	right	precentral	54	4.67	(38,-28,58) (42,-24,46) (34,-16,54)

Connectivity clusters within the hand representation using FWE correction of p = .05 for intensity and an extent threshold of 20 voxels; structural seeds survived an additional intensity correction for multiple comparisons across the nine seeds tested.

**Fig 6:**
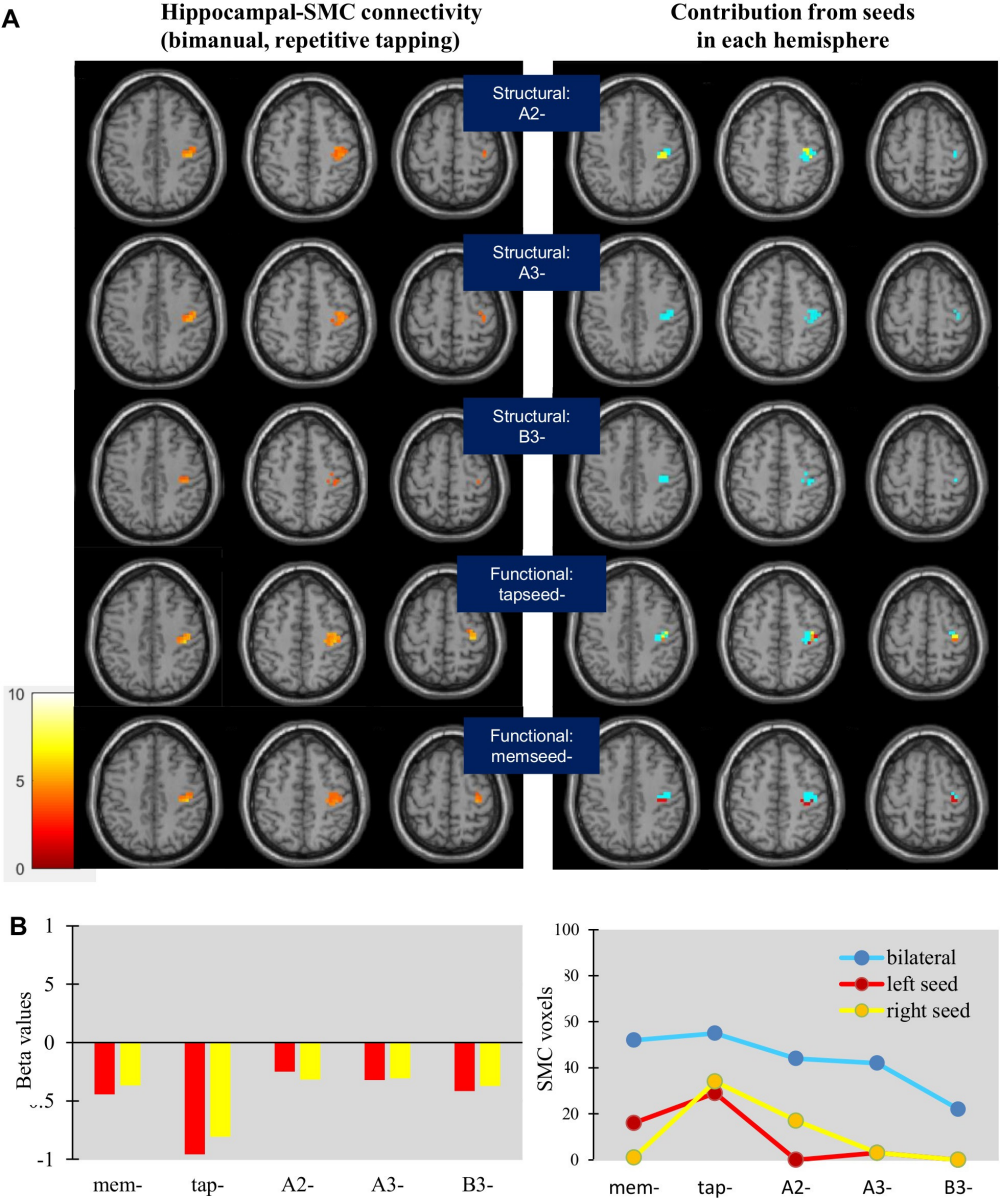
Sensorimotor connectivity of structural and functional seeds during paced repetitive tapping. (A) Negative connectivity generated from structural and functional seeds was observed in the right SMC, almost exclusively from global analysis of the left and right hippocampus. (B) Structural seeds showed greater negative connectivity in the right SMC during repetitive tapping, whereas functional seeds showed greater negative connectivity in the left SMC (light blue) and positive connectivity in the right SMC (magenta) during sequence learning. (C) Beta values indicated equal contributions from the left and right hippocampus (left), with global analysis required to demonstrate most connectivity effects (right).

Negative connectivity limited to the right SMC was demonstrated for the repetitive tapping task; connectivity was most evident through global analysis of both the left and right hippocampal seeds (Fig 6A). Contributions from the left and right hippocampus were roughly equal during repetitive tapping, with the demonstration of connectivity often requiring global analysis (Fig 6B).

### Cortical selectivity for hand representation

Cortical selectivity for the hand representation was evaluated with the hypergeometric distribution to identify the chance probability of the observed number of voxels showing connectivity within the hand representation (Table 5). Connectivity from each seed was selectively restricted to the hand representation.

**Table 5:** Selectivity of task-dependent connectivity for the hand representation.

Task / seed	Left hemisphere			Right hemisphere		
**Sequence learning**	**voxels in SMC**	**voxels in hand**	**p-value**	**voxels in SMC**	**voxels in hand**	**p-value**
**Memseed+**	0	0	--	70	40	0.001
**Memseed-**	396	87	0.001	0	0	--
**Tapseed-**	164	52	0.001	0	0	--
**A1-**	86	23	0.001	0	0	--
**A3-**	53	16	0.001	0	0	--
**B1-**	106	39	0.001	0	0	--
**B2-**	94	45	0.001	0	0	--
**C1-**	45	26	0.001	0	0	--
**Repetitive tapping**						
**Memseed-**	0	0	--	246	52	0.001
**Tapseed-**	0	0	--	451	54	0.001
**A2-**	0	0	--	164	44	0.001
**A3-**	0	0	--	175	42	0.001
**B3-**	0	0	--	71	22	0.001
**total # voxels in population**	548	87	--	564	54	--

For each seed, connectivity was significantly limited to the hand representation; the number of voxels showing connectivity within the SMC and hand representation are reported for each hemisphere. Levels of significance were identified from the hypergeometric distribution.

## Discussion

Connectivity of the hippocampus with sensorimotor cortex (SMC) was examined during two paced motor tasks; behavioral analysis showed that performance during both tasks was under volitional control, although only one task produced learning effects. In both tasks, contributions from the left and right hippocampus often overlapped, jointly generating connectivity in SMC; this connectivity was limited to the hand representation. These findings demonstrate a specific hippocampal influence on the SMC hand representation during volitional movements that does not require motor learning.

### Behavioral analysis

Behavioral analysis of behavior during motor performance addressed two issues: first, whether motor learning occurred across rehearsals, and second, whether volitional control was involved.

Learning effects were evident during sequence learning, with improved precision in intertap intervals and decreased variability across rehearsals. No such effects were evident during repetitive tapping. Learning effects in the sequence learning task occurred over the first 3–4 rehearsals, when cognitive processes are important for skill acquisition [35].

Subjects consistently anticipated the metronome in both tasks. Instructed to press a key in synchrony with the metronome ticks, presented at 500ms intervals, stimulus-driven responses would have followed each metronome tick at a fixed interval. By contrast, the observed anticipatory effect was additive across trials, reflecting subjects’ prior knowledge of tick intervals. In both motor tasks, anticipatory responses thus reflected volitional control.

### Sensorimotor activation

Consistent with previous reports, group analysis showed SMC activation within the omega spur contralateral to the moving hand(s) [36–40].

Under optimal processing parameters, bilateral SMC activation was generated by the bimanual repetitive tapping task. Sequence learning, performed with the right hand only, generated left SMC activation during group analysis, but bilateral activation during individual analysis. This bilateral activation likely reflects inhibitory influences between motor regions in opposite hemispheres [41]. Because movements of individual finger and their representations are not independent [42–43], however, activation must reflect some combination of excitatory and inhibitory processes in both hemispheres.

Global conjunctive analysis included both sequence learning and repetitive tapping, improving sensitivity to common motor effects by accounting for task differences in variability. Global analysis showed bilateral activation.

Using processing parameters more suitable for examining hippocampal activity (i.e., a smaller smoothing kernel), the area of SMC activation for both tasks was more restricted and limited to a single hemisphere; activation was limited to the left SMC for sequence learning and the right SMC for repetitive tapping.

### Hippocampal seeds as sources of sensorimotor connectivity

PPI results demonstrate the influence of one neural system on another (effective connectivity), thus differing from functional connectivity, which shows correlated activity between brain areas without causal inference [23]. The current study was designed to detect hippocampal activity that affects SMC activity more during a motor condition than during passive visual viewing.

Functional and structural seeds both identified hippocampal connectivity with SMC. Structural seeds provided the net effect of connectivity from 8–12 voxels; the connectivity of each voxel was computed, then the mean connectivity of all voxels within the structural seed was calculated. Localized from structural seeds with demonstrable connectivity, functional seeds provided a finer-grain localization of the source of connectivity by accounting for individual variability, and may better reflect hippocampal influences on SMC.

Heterogeneous functionality within the hippocampus was reflected in the pattern of functional seed connectivity during the sequence learning task. One functional seed (memseed-) showed negative connectivity in the left SMC hand representation, representing the moving hand; another (memseed+) showed positive connectivity in right SMC, representing the inactive hand. The cortical target and its relationship to movement thus differed, suggesting functional heterogeneity between these seeds.

Although the sign of connectivity differed for these seeds, this difference is difficult to interpret for at least two reasons. First, the PPI method cannot specify whether the effects on SMC activity results from differential input from the hippocampus across motor conditions, or from interactions between the hippocampus and another input. With inhibitory influences between motor regions in opposite hemispheres [41], an interaction of hippocampal input with SMC input from one hemisphere might, for example, generate the opposite sign of connectivity in the other. Second, finger movements require coordination of multiple processes, including excitatory and inhibitory influences between fingers on the same hand and coordination between flexor and extensor muscle movements [42–43]. The relationship between hippocampal connectivity with these processes is unknown and beyond the scope of this study.

The left and right hippocampus are known to show similar connectivity patterns along its long axis [44], but also laterality differences in hippocampal function [45–46]. Both patterns were observed in this study. Connectivity from the left and right hippocampus generally overlapped such that increased sensitivity often resulted from global analysis of both, as evident during repetitive tapping. For structural seeds, this indicates similar functionality for homologous regions of the hippocampus in each hemisphere, effectively working together. By contrast, the magnitude and area of connectivity generated during sequential learning by the left hippocampus was greater than the right (see Fig 5). This difference in connectivity between tasks could reflect either cognitive differences, or task differences in the hand(s) that moved.

### Hippocampal sensorimotor connectivity

Although hippocampal connectivity with the striatum has been observed during motor learning, suggesting a mnemonic-motor interaction [7, 47, 48], this study is the first to find hippocampal interactions with the motor system independent of its role in memory. By identifying where SMC activity was influenced more by moment-to-moment hippocampal activity during movements than during the passive visual condition, the PPI method of analysis was task-specific for motor function, as the magnitude of connectivity was based on the correlation of SMC activity with this movement-specific term. The demonstration of connectivity with the sensorimotor cortex implicates the hippocampus in volitional movements, rather than just striatal-associated movements derived from habits [19, 20, 49, 50].

### Cortical selectivity: The hand representation

The hippocampus is the likely source of theta EEG rhythms [13, 51], which are conducive to motor performance, learning, working memory, sensorimotor integration, and spatial learning [13–17, 33, 37, 52, 53]. The generation of theta waves raises the possibility that SMC connectivity reflects a large, nonspecific process that incidentally affects many cortical regions involved in task performance.

During both motor tasks, however, hippocampal connectivity with SMC was restricted to its hand representation, indicating this connectivity does *not* represent a widespread, nonspecific influence on sensorimotor cortex. Differences in laterality for negative and positive connectivity during unimanual movements further suggest specificity.

### Functional implications

This study demonstrated hippocampal connectivity restricted to the SMC hand representation during volitional finger movements, even in the absence of motor learning. Nonetheless, known hippocampal functions related to memory and sensorimotor integration might partially explain the results.

Hippocampal connectivity with SMC was observed during both sequence learning and repetitive tapping tasks. Although the latter task did not show motor learning effects, memory for the 500ms pacing interval was still evident from anticipatory movements that preceded the metronome. The hippocampus responds differentially to the intervals between stimuli, at least within a sequence [54–56]; thus, hippocampal connectivity may have relayed its “memory” for temporal intervals. Note that task performance did not *require* memory for the pacing intervals, however, as subjects could have simply responded to the metronome, leaving open the question of how and why hippocampal connectivity was recruited.

The hippocampus has been implicated in sensorimotor integration [13, 57, 58]. During sensorimotor integration, motor responses to sensory stimuli are modified, such as a navigational response to sensory surroundings. In the current study, movements were not constrained by acoustic stimuli, evident from the anticipatory motor responses; however, hippocampal connectivity might have modified somatosensory processing in the postcentral gyrus, promoting the appropriate finger movements. Because sensory feedback is already provided via short fiber tracts that connect postcentral with precentral cortex [21], the hippocampus promoting volitional finger movements via sensorimotor integration would reflect a more direct role in motor control.

Further study will be required to fully delineate the role of hippocampal-SMC connectivity.

## Conclusions

Hippocampus connectivity with the SMC hand representation was evident during volitional, paced finger movements; this connectivity did not require motor learning, but was selective for movements. Connectivity from corresponding regions of the left and right hippocampus overlapped extensively, enhancing their individual effects. The role of this connectivity appears to be linked, directly or indirectly, to the control of volitional movements.

## Supporting information

S1 Fig Structural seeds and voxel locations in the hippocampus. Structural seeds provided the mean connectivity from multiple voxels, each calculated separately.
(TIF)

S2 Fig Functional seed locations. The center locations of functional seeds are shown.
(TIF)

S1 Table Coordinates of sampled voxels in left hippocampus. Labelled voxels in the left hippocampus of the MNI-normalized brain, selected from the aal atlas in the WFU_PickAtlas toolbox for SPM.
(DOCX)

S2 Table Coordinates of sampled voxels in right hippocampus. Labelled voxels in the right hippocampus of the MNI-normalized brain, selected from the aal atlas in the WFU_PickAtlas toolbox for SPM.
(DOCX)

S3 Table Connectivity maxima and minima used to select functional seeds from the sequence learning task. (DOCX)

S4 Table Motor task activation using a whole-brain mask. Activation clusters from global analysis of both motor tasks, applying an intensity threshold of p = 0.05 with a family-wise error correction and extent threshold of 50 voxels.
(DOCX)
