# Supplementary figures and images for: Hippocampal connectivity with sensorimotor cortex during volitional finger movements: Laterality and relationship to motor learning
Source: PLoS One. 2019 Sep 19;14(9):e0222064. doi: 10.1371/journal.pone.0222064 (PMC6752792; doi:10.1371/journal.pone.0222064)

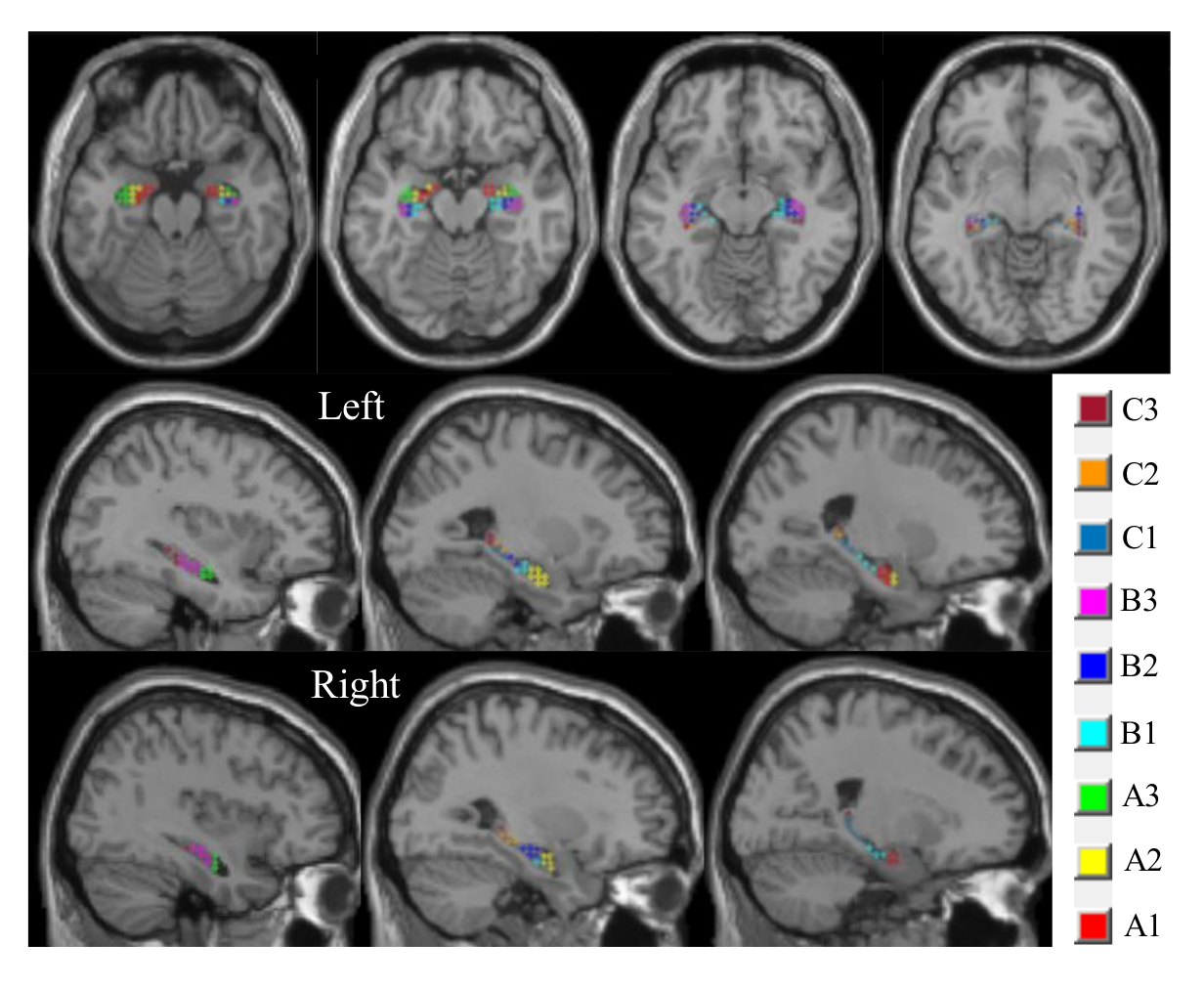

Supplement: S1 Fig — Structural seeds provided the mean connectivity from multiple voxels, each calculated separately. (TIF) [file pone.0222064.s001.tif]

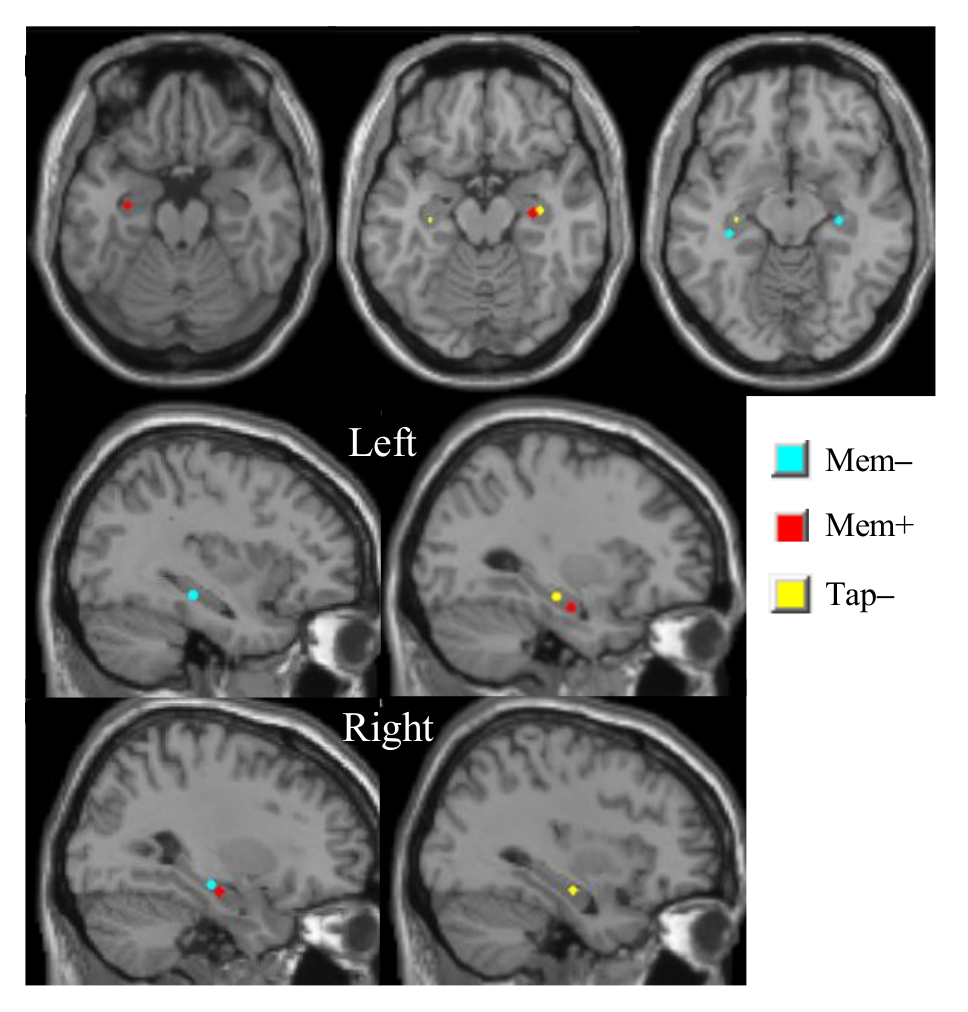

Supplement: S2 Fig — The center locations of functional seeds are shown. (TIF) [file pone.0222064.s002.tif]
