# Supplementary material for: Hippocampal connectivity with sensorimotor cortex during volitional finger movements: Laterality and relationship to motor learning
Source: PLoS One. 2019 Sep 19;14(9):e0222064. doi: 10.1371/journal.pone.0222064 (PMC6752792; doi:10.1371/journal.pone.0222064)
Supplement: S1 Table — Labelled voxels in the left hippocampus of the MNI-normalized brain, selected from the aal atlas in the WFU_PickAtlas toolbox for SPM. (DOCX) [file pone.0222064.s003.docx]

**Table S1. Coordinates of sampled voxels in left hippocampus.**

| **Identifier** | **(X,Y,Z)** | **Identifier** | **(X,Y,Z)** |
| --- | --- | --- | --- |
| L111 | (-14,-40,8) | L112 | (-18,-40,8) |
| L113 | (-22,-40,8) | L121 | (-14,-40,4) |
| L122 | (-18,-40,4) | L123 | (-22,-40,4) |
| L124 | (-26,-40,4) | L131 | (-26,-40,0) |
| L132 | (-30,-40,0) | L211 | (-18,-36,0) |
| L212 | (-22,-36,0) | L213 | (-26,-36,0) |
| L214 | (-30,-36,0) | L221 | (-26,-36,-4) |
| L222 | (-30,-36,-4) | L223 | (-34,-36,-4) |
| L311 | (-18,-32,-4) | L312 | (-22,-32,-4) |
| L313 | (-26,-32,-8) | L321 | (-26,-32,-8) |
| L322 | (-30,-32,-8) | L323 | (-34,-32,-8) |
| L331 | (-30,-32,-12) | L332 | (-34,-32,-12) |
| L411 | (-18,-28,-8) | L412 | (-22,-28,-8) |
| L413 | (-26,-28,-8) | L414 | (-30,-28,-12) |
| L415 | (-34,-28,-8) | L421 | (-18,-28,-12) |
| L422 | (-30,-28,-12) | L423 | (-34,-28,-12) |
| L511 | (-18,-24,-12) | L512 | (-22,-24,-12) |
| L513 | (-26,-24,-12) | L514 | (-30,-24,-12) |
| L515 | (-34,-24,-12) | L516 | (-38,-24,-12) |
| L521 | (-26,-24,-16) | L522 | (-30,-24,-16) |
| L523 | (-34,-24,-16) | L611 | (-26,-20,-12) |
| L612 | (-30,-20,-12) | L613 | (-34,-20,-12) |
| L621 | (-22,-20,-16) | L622 | (-26,-20,-16) |
| L623 | (-30,-20,-16) | L624 | (-34,-20,-16) |
| L711 | (-22,-16,-16) | L712 | (-26,-16,-16) |
| L713 | (-30,-16,-16) | L714 | (-34,-16,-16) |
| L721 | (-22,-16,-20) | L722 | (-26,-16,-20) |
| L723 | (-30,-16,-20) | L724 | (-34,-16,-20) |
| L811 | (-18,-12,-16) | L812 | (-22,-12,-16) |
| L813 | (-26,-12,-16) | L814 | (-30,-12,-16) |
| L815 | (-34,-12,-16) | L821 | (-18,-12,-20) |
| L822 | (-22,-12,-20) | L823 | (-26,-12,-20) |
| L824 | (-30,-12,-20) | L825 | (-34,-12,-20) |
| L831 | (-22,-12,-24) | L832 | (-26,-12,-24) |
| L911 | (-14,-8,-16) | L912 | (-18,-8,-16) |
| L913 | (-30,-8,-16) | L921 | (-14,-8,-20) |
| L922 | (-18,-8,-20) | L923 | (-22,-8,-20) |
| L924 | (-26,-8,-20) | L925 | (-30,-8,-20) |
| L931 | (-22,-8,-24) | L932 | (-26,-8,-24) |

Labelled voxels in the the left hippocampus of the MNI-normalized brain, selected from the aal atlas in the WFU_PickAtlas toolbox for SPM.
