# Supplementary material for: Hippocampal connectivity with sensorimotor cortex during volitional finger movements: Laterality and relationship to motor learning
Source: PLoS One. 2019 Sep 19;14(9):e0222064. doi: 10.1371/journal.pone.0222064 (PMC6752792; doi:10.1371/journal.pone.0222064)
Supplement: S2 Table — Labelled voxels in the right hippocampus of the MNI-normalized brain, selected from the aal atlas in the WFU_PickAtlas toolbox for SPM. (DOCX) [file pone.0222064.s004.docx]

**Table S2. Coordinates of sampled voxels in right hippocampus.**

| **Identifier** | **(X,Y,Z)** | **Identifier** | **(X,Y,Z)** |
| --- | --- | --- | --- |
| R111 | (14,-36,8) | R112 | (18,-36,8) |
| R113 | (22,-36,8) | R121 | (22,-36,4) |
| R122 | (26,-36,4) | R131 | (22,-36,0) |
| R132 | (26,-36,0) | R133 | (30,-36,0) |
| R141 | (26,-36,-4) | R142 | (30,-36,-4) |
| R143 | (36,-36,-4) | R144 | (34,-36,-8) |
| R211 | (18,-32,-4) | R212 | (22,-32,-4) |
| R213 | (26,-32,-4) | R214 | (30,-32,-4) |
| R215 | (34,-32,-4) | R221 | (26,-32,-8) |
| R222 | (30,-32,-8) | R223 | (34,-32,-8) |
| R311 | (22,-28,-8) | R312 | (26,-28,-8) |
| R313 | (30,-28,-8) | R314 | (34,-28,-8) |
| R321 | (34,-28,-8) | R322 | (38,-28,-12) |
| R411 | (34,-24,-8) | R421 | (22,-24,-12) |
| R422 | (26,-24,-12) | R423 | (30,-24,-12) |
| R424 | (34,-24,-12) | R425 | (38,-24,-12) |
| R511 | (34,-20,-8) | R521 | (26,-20,-12) |
| R522 | (30,-20,-12) | R523 | (34,-20,-12) |
| R524 | (38,-20,-12) | R531 | (22,-20,-16) |
| R532 | (26,-20,-16) | R533 | (30,-20,-16) |
| R534 | (34,-20,-16) | R535 | (38,-20,-16) |
| R611 | (26,-16,-12) | R612 | (30,-16,-12) |
| R613 | (34,-16,-12) | R621 | (22,-16,-16) |
| R622 | (26,-16,-16) | R623 | (30,-16,-16) |
| R624 | (34,-16,-16) | R625 | (38,-16,-16) |
| R626 | (42,-16,-16) | R631 | (30,-16,-20) |
| R632 | (34,-16,-20) | R633 | (38,-16,-20) |
| R711 | (18,-12,-16) | R712 | (22,-12,-16) |
| R713 | (22,-12,-16) | R714 | (30,-12,-16) |
| R715 | (30,-12,-16) | R716 | (38,-12,-16) |
| R721 | (22,-12,-20) | R722 | (26,-12,-20) |
| R723 | (30,-12,-20) | R724 | (34,-12,-20) |
| R725 | (38,-12,-20) | R731 | (34,-12,-24) |
| R732 | (38,-12,-24) | R811 | (18,-8,-16) |
| R812 | (22,-8,-16) | R813 | (30,-8,-16) |
| R814 | (34,-8,-16) | R821 | (22,-8,-20) |
| R822 | (26,-8,-20) | R823 | (30,-8,-20) |
| R824 | (34,-8,-20) | R825 | (38,-8,-20) |
| R831 | (30,-8,-24) | R832 | (34,-8,-24) |

Labelled voxels in the right hippocampus of the MNI-normalized brain, selected from the aal atlas in the WFU_PickAtlas toolbox for SPM.
