# Supplementary material for: Hippocampal connectivity with sensorimotor cortex during volitional finger movements: Laterality and relationship to motor learning
Source: PLoS One. 2019 Sep 19;14(9):e0222064. doi: 10.1371/journal.pone.0222064 (PMC6752792; doi:10.1371/journal.pone.0222064)
Supplement: S3 Table — (DOCX) [file pone.0222064.s005.docx]

**Table S3. Connectivity maxima and minima used to select functional seeds from the sequence learning task.**

| *Subject* | *Seed laterality* | *Memseed+* | | | | *Memseed-* | | | |
| --- | --- | --- | --- | --- | --- | --- | --- | --- | --- |
|  |  | *Maximum* | *Hemi* | *Pre/post* | *Hand?* | *Minimum* | *Hemi* | *Pre/post* | *Hand?* |
| 01 | Left | (58,-12,50) | Right | post | no | (-42,-44,62) | Left | post | no |
|  | Right | (42,-20,66) | Right | pre | no | (-42,-40,62) | Left | post | no |
| 02 | Left | (42,-24,62) | Right | pre | no | (-38,-20,66) | Left | pre | no |
|  | Right | (42,-24,62) | Right | pre | no | (-38,-24,70) | Left | pre | no |
| 03 | Left | (46,-36,62) | Right | post | no | (42,-32,62) | Right | post | no |
|  | Right | (46,-28,62) | Right | post | no | (-42,-24,66) | Left | pre | no |
| 04 | Left | (46,-12,54) | Right | pre | no | (26,-40,74) | Right | post | no |
|  | Right | (50,-12,54) | Right | pre | no | (42,-32,62) | Right | post | no |
| 05 | Left | (34,-20,70) | Right | pre | no | (-46,-20,58) | Left | post | no |
|  | Right | (42,-12,66) | Right | pre | no | (-46,-16,58) | Left | post | no |
| 06 | Left | (50,-32,58) | Right | post | no | (-34,-24,70) | Left | pre | no |
|  | Right | (38,-20,66) | Right | pre | no | (-30,-24,70) | Left | pre | no |
| 07 | Left | (34,-24,70) | Right | pre | no | (-30,-20,70) | Left | pre | no |
|  | Right | (34,-24,54) | Right | pre | yes | (-34,-20,70) | Left | pre | no |
| 08 | Left | (42,-24,66) | Right | pre | no | (26,-36,74) | Right | post | no |
|  | Right | (-38,-20,66) | Left | pre | no | (-34,-40,66) | Left | post | no |
| 09 | Left | (34,-20,50) | Right | pre | yes | (-34,-36,66) | Left | post | no |
|  | Right | (-38,-36,62) | Left | post | yes | (-34,-36,66) | Left | post | no |
| 10 | Left | (38,-20,66) | Right | pre | no | (-46,-32,58) | Left | post | yes |
|  | Right | (38,-20,66) | Right | pre | no | (-46,-32,58) | Left | post | yes |
| 11 | Left | (50,-8,50) | Right | pre | no | (-34,-40,66) | Left | post | no |
|  | Right | (-34,-36,66) | Left | post | no | (-34,-40,66) | Left | post | no |
| 12 | Left | (38,-24,62) | Right | pre | yes | (54,-24,50) | Right | post | no |
|  | Right | (38,-24,62) | Right | pre | yes | (54,-24,50) | Right | post | no |
| 13 | Left | (34,-28,58) | Right | pre | yes | (42,-24,62) | Right | pre | no |
|  | Right | (54,-28,54) | Right | post | no | (34,-28,70) | Right | pre | no |
