# Supplementary material for: Hippocampal connectivity with sensorimotor cortex during volitional finger movements: Laterality and relationship to motor learning
Source: PLoS One. 2019 Sep 19;14(9):e0222064. doi: 10.1371/journal.pone.0222064 (PMC6752792; doi:10.1371/journal.pone.0222064)
Supplement: S4 Table — Activation clusters from global analysis of both motor tasks, applying an intensity threshold of p = 0.05 with a family-wise error correction and extent threshold of 50 voxels. (DOCX) [file pone.0222064.s006.docx]

**Table S4. Motor task activation using a whole-brain mask.**

| **Hemisphere** | **Region** | **Cluster size** | **Z-score**  **(peak)** | **Peak Coordinates** |
| --- | --- | --- | --- | --- |
| Left | postcentral / precentral / inferior parietal / inferior frontal | 284 | 5.89 | (-46,-36,50) |
|  | superior temporal gyrus |  | 5.68  5.65 | (-50,4,-6)  (-42,-4,46) |
| Bilateral | supplementary motor cortex | 59 | 5.82 | (-2,4,66) |
| Left | superior temporal gyrus | 81 | 5.51  5.28 | (-54,-36,14)  (-58,-36,14) |
| Right | postcentral / precentral / inferior parietal | 163 | 5.39  5.28  5.05 | (38,-52,42)  (50,-36,50)  (46,-16,50) |
| Bilateral | cerebellum | 55 | 5.30  4.96 | (-2,-60,-22)  (-18,-56,-26) |

Activation clusters from global analysis of both motor tasks, applying an intensity threshold of p=0.05 with a family-wise error correction and extent threshold of 50 voxels.
